# Loss of the SV2-like Protein SVOP Produces No Apparent Deficits in Laboratory Mice

**DOI:** 10.1371/journal.pone.0068215

**Published:** 2013-07-24

**Authors:** Jia Yao, Horacio O. de la Iglesia, Sandra M. Bajjalieh

**Affiliations:** 1 Department of Pharmacology, University of Washington, Seattle, Washington, United States of America; 2 Department of Biology, University of Washington, Seattle, Washington, United States of America; University of Leicester, United Kingdom

## Abstract

Neurons express two families of transporter-like proteins − Synaptic Vesicle protein 2 (SV2A, B, and C) and SV2-related proteins (SVOP and SVOPL). Both families share structural similarity with the Major Facilitator (MF) family of transporters. SV2 is present in all neurons and endocrine cells, consistent with it playing a key role in regulated exocytosis. Like SV2, SVOP is expressed in all brain regions, with highest levels in cerebellum, hindbrain and pineal gland. Furthermore, SVOP is expressed earlier in development than SV2 and is one of the neuronal proteins whose expression declines most during aging. Although SV2 is essential for survival, it is not required for development. Because significant levels of neurotransmission remain in the absence of SV2 it has been proposed that SVOP performs a function similar to that of SV2 that mitigates the phenotype of SV2 knockout mice. To test this, we generated SVOP knockout mice and SVOP/SV2A/SV2B triple knockout mice. Mice lacking SVOP are viable, fertile and phenotypically normal. Measures of neurotransmission and behaviors dependent on the cerebellum and pineal gland revealed no measurable phenotype. SVOP/SV2A/SV2B triple knockout mice did not display a phenotype more severe than mice harboring the SV2A/SV2B gene deletions. These findings support the interpretation that SVOP performs a unique, though subtle, function that is not necessary for survival under normal conditions.

## Introduction

Synaptic vesicles are a specialized class of recycling endosome that mediate the release of neurotransmitters. They contain the proteins required to fill with neurotransmitter, target to active zones at the presynaptic membrane and fuse in response to elevations in cytoplasmic calcium. Among the proteins specific to synaptic vesicles are two transporter-like proteins termed synaptic vesicle protein 2 (SV2) [1-3] and SV2-related protein (SVOP) [4].

There are three isoforms of SV2 in mammals (SV2A, B, and C) and two SVOPs (SVOP and SVOPL). All contain structural features of the Major Facilitator (MF) transporter superfamily [5]. Phylogenetic analysis of the amino acid sequences of MF proteins places SVOP and SV2 most closely to organic ion transporters [6]. While it has been suggested that SV2s are calcium transporters [7] and neurons lacking SV2B were reported to have elevated cytoplasmic calcium [8], synaptic vesicles lacking SV2 do not demonstrate altered calcium uptake (R. Bartlett and S. Bajjalieh, unpublished observations). Both SV2 and SVOP bind nucleotides, though neither acts as a nucleotide transporter [9,10]. To date, no transport substrate has been confirmed for either SV2 or SVOP.

SV2 is essential for survival [7,11]. Mice lacking the major SV2 isoform, SV2A, experience severe seizures beginning at postnatal day seven, and die within three weeks of birth. SV2A is the binding site of the antiepileptic drugs represented by levetiracetam (Keppra^TM^) [12]. Because they are the target of this new class drugs, the search for the molecular actions of SV2 and related proteins has garnered a new sense of urgency.

Reverse genetic studies indicate that the SV2s are positive modulators of evoked vesicle fusion. Loss of SV2A or SV2A and SV2B reduces synaptic release probability [8,11,13,14] due to a reduction in the readily releasable pool of vesicles [15,16]. Loss of SV2 does not, however, prevent all neurotransmission. It has therefore been hypothesized that an SV2-dependent function essential for neurotransmission might be supplied by the related protein SVOP.

SVOP was identified in an *in silico* screen for SV2 homologs and it shares 20–22% sequence identity with SV2 [4]. SVOP is expressed early in the developing nervous system, in contrast to SV2, which is expressed at lower levels before birth [4,17]. This suggests that SVOP may contribute to neuronal development. SVOP may also play a role in brain aging. A transcriptome analysis of human brain identified SVOP as the gene whose expression decreases the most with aging [18]. Likewise, RNA microarray studies found that SVOP expression is decreased in older rats [19].

In an effort to elucidate the function of SVOP, we generated mice lacking SVOP and examined their phenotypes. We also created SVOP/SV2A/SV2B triple knockout mice to determine if the absence of all three proteins would reveal a function essential to development or neurotransmission.

## Materials and Methods

### Conditional targeting of the SVOP gene

Animal studies were performed in compliance with the National Institutes of Health Guide for the Care and Use of Laboratory Animals. The animal protocol was reviewed and approved by the Institutional Animal Care and Use Committee of the University of Washington.

**Figure 1:**
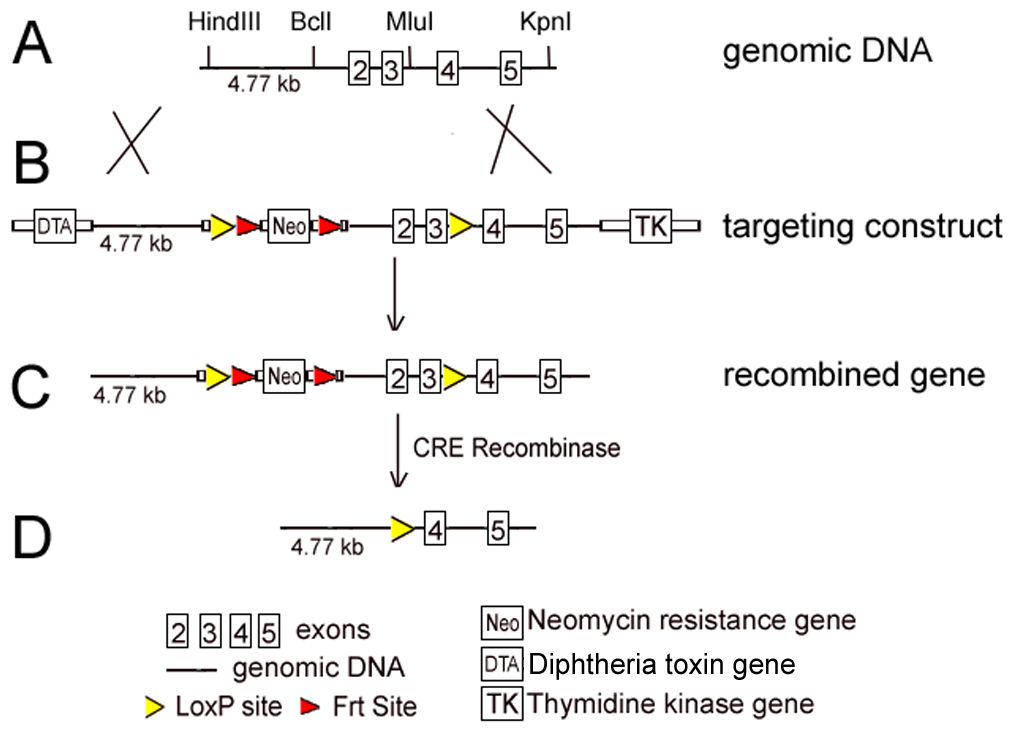
Targeted disruption of the SVOP gene. Shown is the strategy used to generate mice lacking SVOP. **A)** An 11.4kb genomic DNA containing exons 2-5 of the SVOP gene was used for generating a targeting construct. **B)** A targeting construct was generated in which exons 2 and 3 were flanked by Cre recombinase recognition (*loxP*) sites. A cDNA encoding neomycin resistance protein was included to allow screening of embryonic stem cells. To allow removal of the neomycin resistant gene, it was flanked by Flipper recombinase recognition (Frt) sites. **C)** Map of the targeted gene generated by homologous recombination. **D)** Upon Cre recombinase-induced excision, exons 2 and 3 are removed. This results in a truncated protein after the codon encoding a.a.18.

A BAC clone containing the entire SVOP gene from a C57BL/6 mouse genomic library was obtained from BACPAC Resources Center. An 11.4 kb HindIII –KpnI fragment containing exons 2–5 was isolated. This fragment was subcloned into pBluescript II KS(-) to construct a conditional gene targeting vector for SVOP as depicted in Figure 1. A neomycin resistance gene flanked by two Frt sites was inserted in front of exon 2. Cre recombinase sites were inserted 5’ to the Frt-neomycin cassette and after exon 3. cDNAs encoding diphtheria toxin (DTA) and thymidine kinase (TK) were placed at the 5’ and 3’ ends of the targeting fragment for selection against non-homologous recombination. G4 cells, a 129Sv × C57BL/6 F1 hybrid ES cell line [20], were transfected with the linearized targeting construct and selected for homologous recombination using G418 and ganciclovir. Resistant colonies were screened by Southern blot and PCR for homologous recombination. One clone carrying the conditional targeted SVOP gene was injected into C57BL/6 blastocysts and implanted into pseudo pregnant females. One of two chimeric males produced offspring heterozygous for the targeted SVOP gene. Heterozygous mice were used for interbreeding to produce homozygous (SVOP^Flox/Flox^) and heterozygous (SVOP^WT/Flox^) as well as wild-type (SVOP^WT/WT^) mice.

### Generation of SVOP knockout mice

Male mice homozygous for the targeted SVOP gene (SVOP ^Flox/Flox^) were crossed to Mox2^Cre/+^ transgenic females, which express Cre recombinase under the control of the ubiquitous Mox2 promoter, including in germline cells [21] (Generously provided by Dr Richard Palmiter at the University of Washington). Crossing SVOP^Flox/Flox^ mice with Mox2^Cre/+^ transgenic mice resulted in deletion of exons 2 and 3 in the SVOP gene. The resulting mRNA encodes a protein that is truncated at a.a. 18. Offspring carrying the Cre gene were heterozygous for the SVOP gene disruption (SVOP ^+/-^Mox2 ^Cre/+^). These mice were bred with wild type mice to remove Mox2-Cre gene. SVOP ^+/-^ mice are bred to produce homozygous (SVOP^-/-^), heterozygous (SVOP^+/-^) as well as wild-type (SVOP^+/+^) mouse colonies.

### Primary neuronal cell culture

Isolated primary hippocampal autaptic neurons cultured on astrocyte microislands were used for electrophysiological recordings [16]. Briefly, hippocampi from mice homozygous for the targeted SVOP gene or wild type mice were dissected from the brains of postnatal day 0 or 1 (P0 or P1) pups, dissociated by papain digestion, and plated on astrocyte microislands. The hippocampal neurons were maintained in Eagle’s minimal essential medium supplemented with 10% horse serum, 1% N2 supplement, 20 mM glucose, 2 mM glutamax, 25 mM HEPES, 50U/ml penicillin, and 50µg/ml streptomycin. Hippocampal cultures were infected before 3 days in vitro (DIV) with lenti virions encoding Cre-eGFP or eGFP. Electrophysiological recordings were performed on DIV 14–20 cultures. Neurons were selected for recording if they displayed eGFP fluorescence indicating expression of the inserted gene(s). A total of ten cultures were used for the reported electrophysiological experiments.

Conventional cultures of hippocampal neuron were prepared on six-well plates pre-coated with poly-d-lysine. Neurons were maintained and infected with lenti virions as described for autapse cultures. On DIV 11-14, cultures were rinsed two times with cold PBS and harvested in Solubilization Buffer (150 mM NaCl, 50 mM Tris-HCl, pH 7.4, 1 mM EDTA, 1% NP-40, 1× protease inhibitor mixture). Samples were prepared for standard SDS-PAGE and subjected to Western blot analyses. The efficiency of SVOP knockdown by Cre was measured using a polyclonal antibody against SVOP. Expression levels of SV2 and synaptotagmin were detected with a monoclonal anti-SV2 [1] and a polyclonal anti-synaptotagmin antibody [22] respectively. Neuron-specific β3-tubulin (Covance) was probed for as a loading control.

### Electrophysiological recordings

Isolated neurons were subjected to whole-cell voltage-clamp recording. Data were acquired using an EPic-9 amplifier (HEKA Elektronik, Lambrecht, Germany). Recording electrodes were typically 2.5–3.5 MΩ, and series resistance was compensated up to 70% with 10 µs feedback compensation. Cells with series resistance >20 MΩ (before series resistance compensation) or leak currents >250 pA were excluded from analyses. All experiments were performed at 21^°^C in a temperature-controlled chamber. The extracellular solution consisted of 119 mM NaCl, 5 mM KCl, 2.5 mM CaCl_2_, 1.5 mM MgCl_2_, 20 mM HEPES, 30 mM glucose, and 0.01 mM glycine, pH 7.3 (310 mOsm). The pipette solution contained the following: 131 mM K-gluconate, 17.5 mM KCl, 9 mM NaCl, 1 mM MgCl_2_, 10 mM HEPES, and 0.2 mM EGTA, pH 7.2 (328 mOsm). Cells were held at −60 mV and stimulated with a 1 ms depolarization to +20 mV to evoke neurotransmitter release. Excitatory post synaptic currents (EPSCs) were acquired with Pulse software (HEKA Elektronik) and reported as the mean ± SEM.

To measure spontaneous miniature excitatory postsynaptic current (mEPSC), neurons were held at −60 mV and spontaneous events were monitored during a 5 min recording period. The amplitude and frequency of mEPSCs were analyzed with Mini Analysis software (Synaptosoft, Decatur, GA).

### Protein expression

Brains from littermate pups of SVOP^+/-^crosses were quickly removed and homogenized in buffered sucrose solution supplemented with a protease inhibitor cocktail (Halt Protease Inhibitor Cocktail, Thermo Scientific). After centrifugation at 1300×g for 10min, postnuclear supernatants (PNS) were collected and used for Western blot analysis. Equal amounts of protein were separated by SDS-PAGE and transferred to polyvinylidene difluoride (PVDF) membranes. Blots were probed for the indicated proteins. Antibody binding was detected by chemiluminescence and imaged using a Kodak Image Station 440. Actin signals in each lane were used to normalize signals to total protein loading. Antibodies used include: a monoclonal antibody against synaptophysin (Millipore), a monoclonal antibody against VGlut1 (Millipore), a monoclonal antibody against Vesicle Associated Membrane Protein 2 (Vamp2) (Synaptic Systems), a polyclonal antibody against the vesicular proton ATPase (116 kDa subunit) (Synaptic Systems), a monoclonal antibody against α –adaptin (Sigma), a polyclonal antibody against clathrin heavy chain (Abcam), a monoclonal antibody against actin (Sigma), a monoclonal antibody against SV2 [1], a polyclonal antibody against synaptotagmin [22], and a polyclonal antibody generated against a peptide corresponding to a.a. 8- 22 of SVOP.

### Rotarod test

Twelve SVOP knockout mice (SVOP^-/-^) and 12 wild type (SVOP^+/+^) littermates at 8-10 weeks of age were used in this analysis of motor coordination. Within each genotype, 6 males and 6 females were included. The mice were housed in a temperature and humidity-controlled facility on a 12h: 12h light: dark cycle and tested near the end of the light phase. All mice were transported to the testing room 15 min prior to the beginning of experiment to allow them to habituate to the environment.

A Rotamex4/8 apparatus (Columbus Instruments, Columbus, OH) was used for testing. Mice were placed on a rod that accelerated smoothly from 4 rpm to 40 rpm over a 5 min period. The latency to fall or the latency to cling to the rod and passively rotate was recorded. Animals were given three trials per day for 4 consecutive days. Animals were allowed to rest at least 10 min between trials to avoid fatigue.

### Circadian behavioral studies

Six SVOP^-/-^ mice and six SVOP^+/+^ littermates at 9 weeks of age were included in circadian behavioral studies. Within each genotype, 3 males and 3 females were included. Mice were individually housed in cages, and food and water were available *ad lib*. Cages were equipped with a running wheel and kept in a ventilated, light-tight chamber. Wheel-running activity was monitored continuously using Clocklab software (Actimetrics) and data were collected in 10-min bins. Mice were housed under a 12:12 LD (12 h light : 12 h dark) cycle (lights on 7: 00 AM PST) with 200-lux intensity for 12 d before release into constant darkness (DD) for 14 d. Free-running periods during DD cycles were determined using periodogram analysis with the El Temps software (Diéz-Noguera, University of Barcelona, Barcelona, Spain).

## Results

### Generation of SVOP conditional knockout mice

Because SVOP is expressed early in development [4,17], disruption of the SVOP gene had the potential to produce an embryonic lethal phenotype. To overcome this, we generated a conditional SVOP knockout line, placing *loxP* sites in the introns preceding exon 2 and after exon 3 in the SVOP gene. Excision of this portion of the gene by Cre recombinase produces a frame shift and, in turn, a truncated protein. Breeding mice heterozygous for a floxed SVOP gene produced homozygous (SVOP^Flox/Flox^) and heterozygous (SVOP^WT/Flox^) as well as wild-type (SVOP^WT/WT^) mice at near Mendelian frequencies (71:146:64), indicating that insertion of *loxP* sites did not affect viability or development. Western blotting of brain samples from the three genotypes of mice detected similar levels of SVOP, indicating insertion of *loxP* sites did not affect SVOP gene expression (not shown).

### Loss of SVOP does not affect spontaneous or evoked neurotransmission

**Figure 2:**
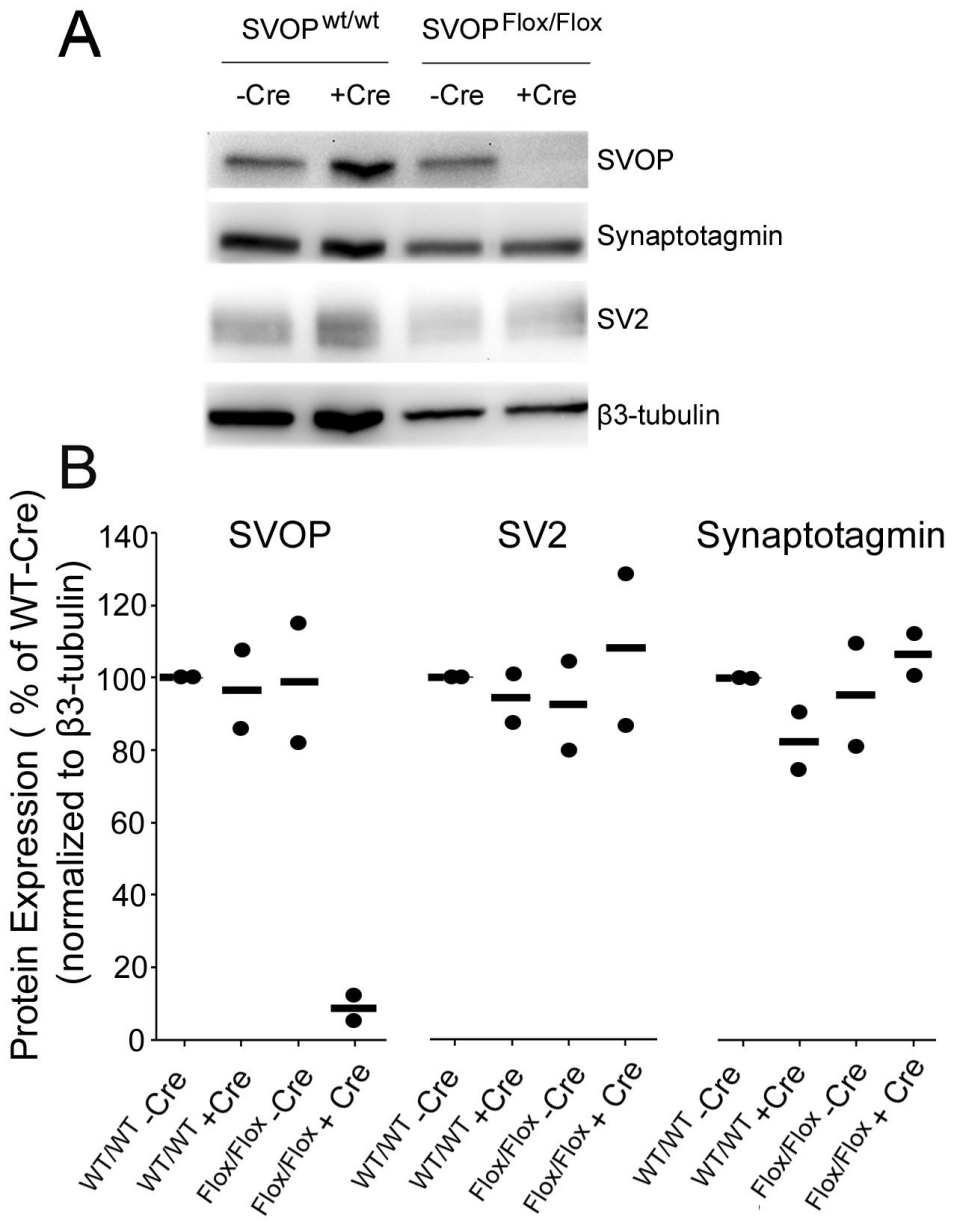
Cre recombinase disrupts SVOP expression in neurons from mice homozygous for the floxed SVOP gene. **A**) Shown are representative Western blots of protein expression in primary hippocampal neurons cultured from newborn pups homozygous for the targeted SVOP gene, or from wild type pups. Neurons were infected with lenti virions encoding Cre-eGFP or eGFP. The expression level of SVOP and the indicated proteins were detected by Western blot analysis. β3-tubulin, a neuron-specific isoform of tubulin, was probed for as a loading control. **B**) Plots of Western blot results from two independent experiments. Each dot represents one datum, and a horizontal line indicates the mean. The signals for SVOP, SV2 and synaptotagmin were normalized to β3-tubulin in the same lane and expressed as a percentage of the wild type - Cre lane in the same blot. SVOP was dramatically decreased in SVOP^Flox/Flox^ neurons expressing Cre. Expression of the synaptic vesicle proteins synaptotagmin and SV2 was similar across conditions.

To assess the role of SVOP in synaptic transmission, we compared spontaneous and evoked release in isolated autaptic neurons from wild-type mice and from SVOP^Flox/Flox^ mice in which SVOP was knocked out by expression of Cre recombinase. Cultures from wild type and SVOP^Flox/Flox^ mice were infected with lenti virions expressing either Cre-eGFP fused to a nuclear localization sequence (NLS) or eGFP alone. The lentiviral expression system achieved more than 95% infection efficiency, as judged by the presence of green fluorescence in neurons. As shown in Figure **2**, expression of Cre-eGFP disrupted SVOP expression in cultured neurons from SVOP^Flox/Flox^ mice but not SVOP^WT/WT^ mice. Expression of eGFP did not alter SVOP expression in either type of neuron. Expression of the synaptic vesicle proteins SV2 and synaptotagmin did not differ between cultures, suggesting that the effect was specific to expression of SVOP.

Whole-cell voltage-clamp recordings were performed to assess neurotransmission in the presence and absence of SVOP. Neurons were selected for recording based on the intensity of eGFP fluorescence with preference given to brighter cells. To assess quantal size and spontaneous neurotransmission, we measured the amplitude and frequency of miniature excitatory post-synaptic currents (mEPSC).

**Figure 3:**
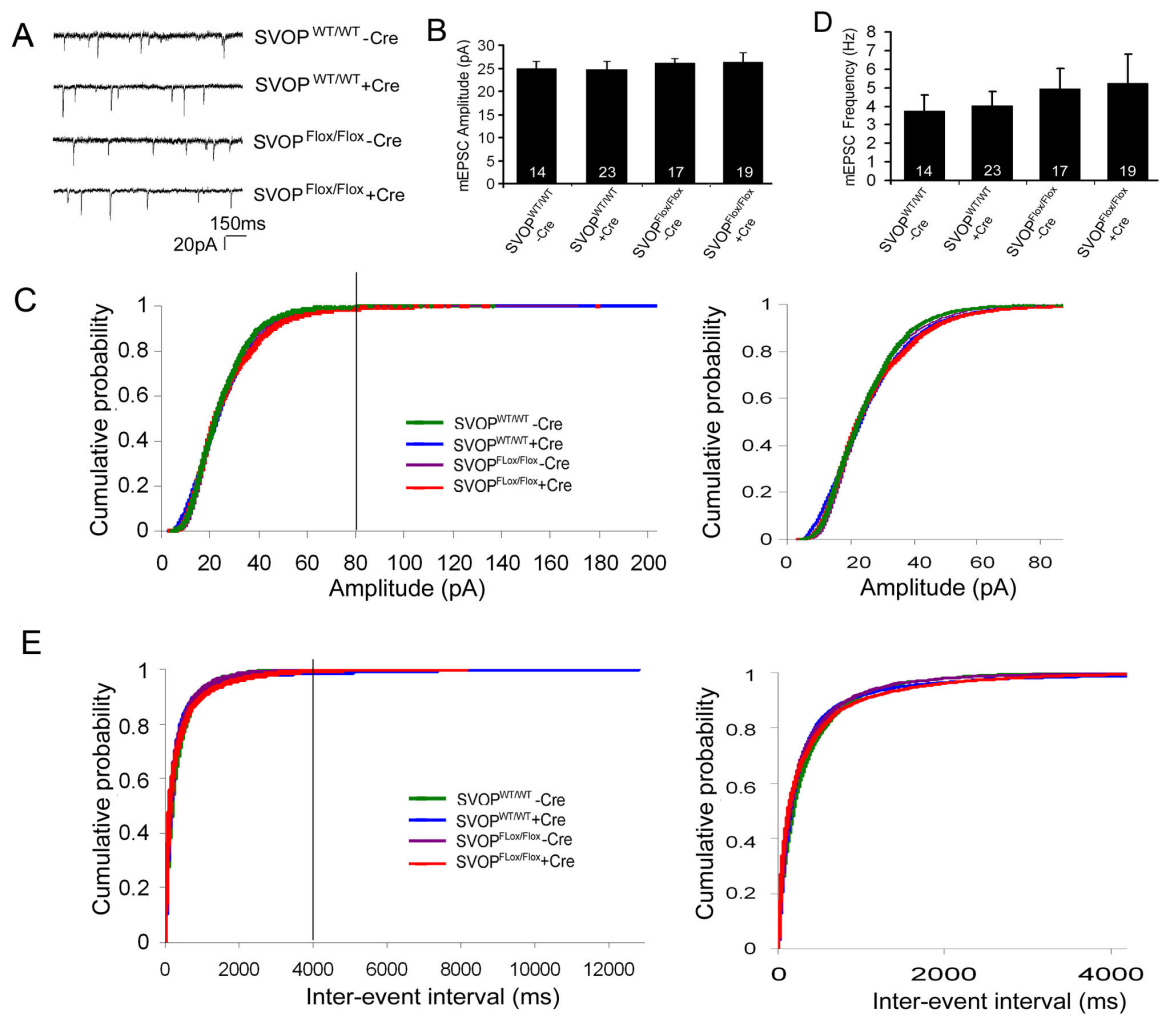
Loss of SVOP does not affect spontaneous neurotransmitter release. Shown are graphs summarizing peak amplitude and frequency of mEPSC. Autaptic hippocampal neurons cultured from mice homozygous for Floxed SVOP genes or wild type littermate controls were infected with lenti virions encoding Cre-eGFP or eGFP. Neurons were analyzed in the whole-cell voltage-clamp configuration at DIV 14-20. Cells were selected for recording according to green fluorescence expression with preference given to brighter cells. Neurons were held at −60 mV, and mini EPSC were recorded in 5 min epochs. Graphs show the mean ± SEM. The number of cells analyzed is indicated within each column. Data are from three different cultures. **A)** Representative traces of mini EPSCs recorded from autaptic hippocampal neurons. **B)** Mean amplitudes of mEPSCs were unchanged across four experimental groups (SVOP^WT/WT^ - Cre, 24.78 ± 1.56 pA; SVOP^WT/WT^ + Cre, 24.74 ± 1.73 pA, SVOP^Flox/Flox^ -Cre 26.13 ± 1.10 pA and SVOP^Flox/Flox^ + Cre 26.28 ± 2.03 pA, *p* = 0.88, two-way ANOVA). **C)** Cumulative probability plots of mEPSC amplitude. The left panel shows the full amplitude range, the right panel is a plot of events with amplitudes < 80 pA. **D)** No differences in mEPSC frequency were observed across all experimental groups (SVOP^WT/WT^ - Cre, 3.76 ± 0.83 Hz; SVOP^WT/WT^ + Cre, 4.00 ± 0.77 Hz, SVOP^Flox/Flox^ -Cre 4.95 ± 1.06 Hz and SVOP^Flox/Flox^ + Cre 5.24 ± 1.58 Hz, *p* = 0.76, two-way ANOVA). **E)** Cumulative probability plots of mEPSC inter-event interval of 4 experimental groups. The left panel is a plot of the full inter-event interval range, the right panel of inter-event intervals <4000ms.

mEPSC amplitude serves as an indicator of quantal size and thus of vesicular transmitter content. Analysis of variance (ANOVA) between the four groups revealed no significant difference in average mEPSC amplitude (Figure 3A-B). Pairwise comparison of cumulative frequency distributions using the Kolmogorov Smirnov test (K–S test) produced *p* values <0.001 suggesting a significant difference between neurons lacking SVOP and controls. However, the *D* values (maximum vertical deviation between the two curves) were small (ranging from 0.0483 to 0.0921) and visual inspection did not reveal a consistent difference in the distributions (Figure 3C). This suggests that the statistical difference does not reflect a biologically relevant difference in vesicle transmitter content. These results indicate that vesicular transmitter content is not altered in the absence of SVOP, and therefore SVOP is neither a neurotransmitter transporter nor a co-factor for the vesicular glutamate transporters.

To assess the role of SVOP in release machinery competence and regulation we measured mEPSC frequency. Average mEPSC frequency was slightly higher in neurons from SVOP^Flox/Flox^ mice than SVOP^WT/WT^ mice, but the difference was not significant (ANOVA, p=0.755) (Figure 3D). Pair-wise comparison of frequency distributions using the K-S test generated significant *p* values (all < 0.01 with one exception in which *p*= 0.07244). As with mEPSC amplitude, however the *D* values were low (ranging from 0.0295 to 0.098), and visual inspection of the cumulative frequency plots revealed no consistent shift (Figure 3E), suggesting there was no biologically relevant change in mEPSC frequency in the absence of SVOP. The normal frequency of mEPSCs indicates that SVOP is not required for vesicle fusion, nor does it regulate non-evoked release.

**Figure 4:**
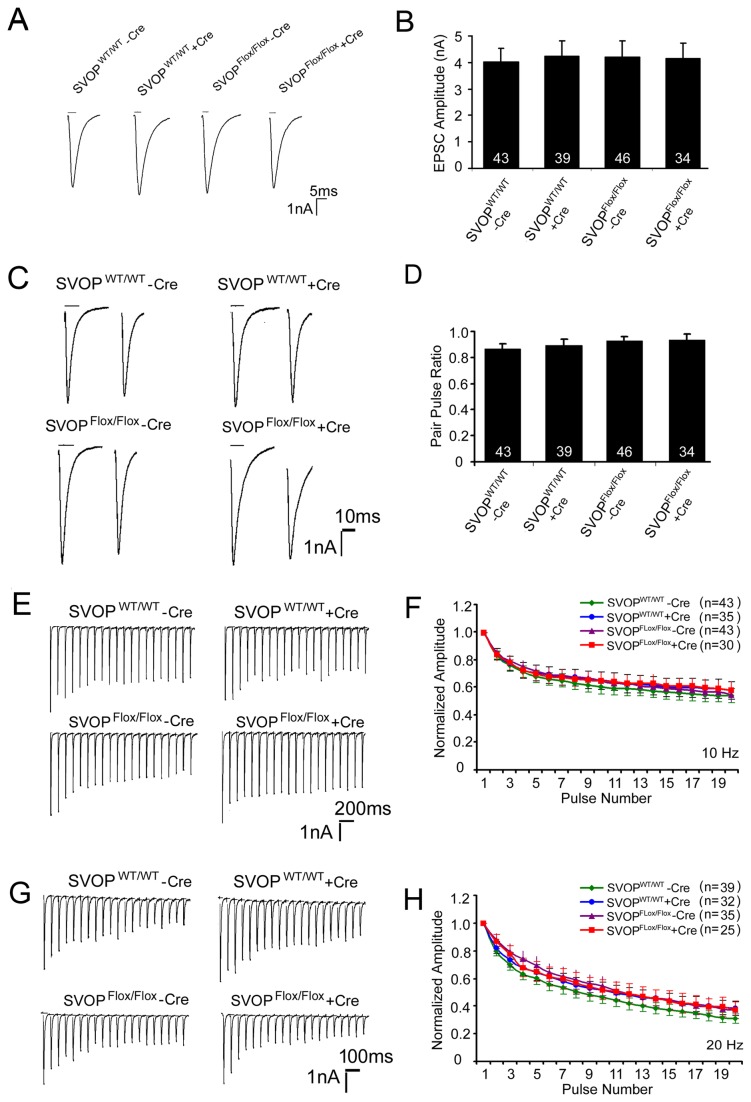
Loss of SVOP has no effect on action-potential evoked transmitter release or short-term plasticity. Neuronal cultures were generated and infected as described in Figure **3**. **A)** Representative traces of single EPSCs. Neurons were held at −60 mV, and single EPSCs were evoked by depolarizing to +20mv for 1 ms. **B)** Average EPSC peak amplitudes of each experimental group. Data are presented as mean ± SEM. The numbers of cells analyzed are indicated within each column. There was no significant difference in the peak amplitude of EPSC between experimental groups (SVOP^WT/WT^ - Cre, 4.03 ± 0.51 nA; SVOP^WT/WT^ + Cre, 4.24 ± 0.58 nA, SVOP^Flox/Flox^ -Cre 4.20 ± 0.60 nA and SVOP^Flox/Flox^ + Cre 4.15 ± 0.58 nA, *p* = 0.99, two-way ANOVA). **C)** Representative traces of paired responses. Autaptic neurons were stimulated with two 1ms pulses separated by 45 ms. **D)** The paired-pulse ratio (PPR) (EPSC peak amplitude response 2/ response 1) was calculated for each cell. The graph shows the mean ± SEM for each experimental group. All experimental groups showed synaptic depression with a mean PPR of less than 1. The mean PPR across experimental groups was indistinguishable (*p* = 0.71, two-way ANOVA). **E)** Representative traces of EPSC in response to stimulus trains delivered at a frequency of 10 Hz. **F)** Shown are mean normalized EPSC amplitudes. Neurotransmission depressed at comparable rates in neurons from all experimental groups. Error bars represent the SEM for each point. The number of cells analyzed is indicated in parentheses. Data are from 10 different cultures. **G**, **H**) 20 Hz stimulus trains produced similar synaptic depression across groups.

To assess the role of SVOP in action potential-evoked neurotransmission, we compared EPSCs evoked by 1 ms depolarizing stimuli. No significant difference in peak EPSC amplitude was found (Figure 4A and 4B), indicating that SVOP is not essential for evoked release. To further investigate the effect of loss of SVOP, we compared two measures of short-term synaptic plasticity that have been shown to reflect synaptic release probability [23,24]. Paired-pulse ratio (PPR), which is the amplitude of a second EPSC divided by the amplitude of the first, was not affected by SVOP gene disruption (Figure 4C and 4D). Likewise, loss of SVOP also did not affect the response to a train of stimuli, which elicits synaptic depression in normal hippocampal neurons. Responses to 20 pulse stimulus trains of 10 or 20 Hz depressed equally across genotypes in the absence and presence of Cre (Figure 4E–H). These results indicate that loss of SVOP does not alter short-term synaptic plasticity and thus synaptic release probability. This contrasts to the SV2 genes, which are required for normal release probability [8,15,16].

### SVOP knockout mice are viable and phenotypically normal

**Figure 5:**
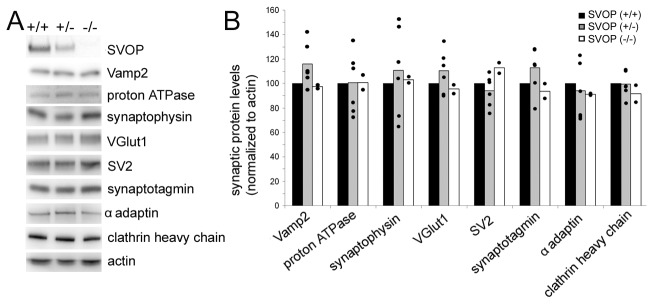
Disruption of the SVOP gene does not change the expression of other synaptic proteins. **A)** Shown are representative Western blot analyses of brain post nuclear supernatant isolated from littermate mice of the indicated genotype. The linear range was determined for each antibody used in Western blot analyses. The amount of protein loaded for each analysis fell within the linear range. Specifically, 5µg of protein were used for detecting SV2, α –adaptin, proton ATPase and VGlut1; 2.5µg of protein were used for detecting synaptophysin and synaptotagmin; 1.5µg of protein were used for detecting Vamp2 and clathrin heavy chain. The antibody used to detect SVOP is derived against a peptide in the amino terminus of SVOP. **B)** Quantification of synaptic protein levels normalized to the amount of actin in the same lane. Brain tissue from 4 wild type, 6 heterozygous and 2 homozygous pups was analyzed. The histograms represent the mean value of expression normalized to wild type values in the same blot. Each dot represents a single datum. Loss of SVOP did not significantly alter expression levels of other synaptic vesicle proteins including Vamp2, the proton ATPase, synaptophysin, VGlut1, SV2, synaptotagmin as well as endocytic proteins such as α adaptin and clathrin heavy chain.

To analyze the role of SVOP in development, we generated SVOP null mice as described in Materials and Methods. Pups from SVOP^+/-^ breeders were born in the expected Mendelian ratios (+/+: +/-: -/- at 56:129: 65). Western blot analysis revealed loss of SVOP protein expression (Figure 5A). SVOP knockout mice were viable and fertile and did not show any overt abnormalities. Unlike mice lacking SV2A, SVOP^-/-^ mice did not demonstrate spontaneous seizures. Thus, SVOP is not essential for normal development or life under standard mouse colony conditions.

### Loss of SVOP does not affect synaptic vesicle protein content

There is accumulating evidence that synaptic vesicle proteins regulate each other’s expression and trafficking to vesicles. For example, loss of SV2 produces a ~45% decrease in total brain levels of synaptotagmin and an 85% decrease in the levels of synaptotagmin in synaptic vesicles [25]. Likewise, disruption of the synaptophysin gene causes a moderate but statistically significant decrease in total levels of Vamp2/synaptobrevin II [26]. To determine if SVOP regulates the expression or trafficking of other synaptic proteins, we examined the expression levels of eight synaptic proteins by Western blot analysis. Actin was used as loading control and protein expression levels were normalized to the level of actin in the same lane. As shown in Figure **5**, loss of SVOP produced no change in the expression of Vamp2, proton ATPase, synaptophysin, VGlut1, SV2, synaptotagmin, α adaptin, and clathrin heavy chain.

### Loss of SVOP does not affect motor coordination or circadian behavior

Surveys of SVOP mRNA revealed that it is expressed at highest levels in the cerebellum, hindbrain and pineal gland [6]. We therefore reasoned that loss of SVOP would be most likely to affect functions controlled by these brains regions.

**Figure 6:**
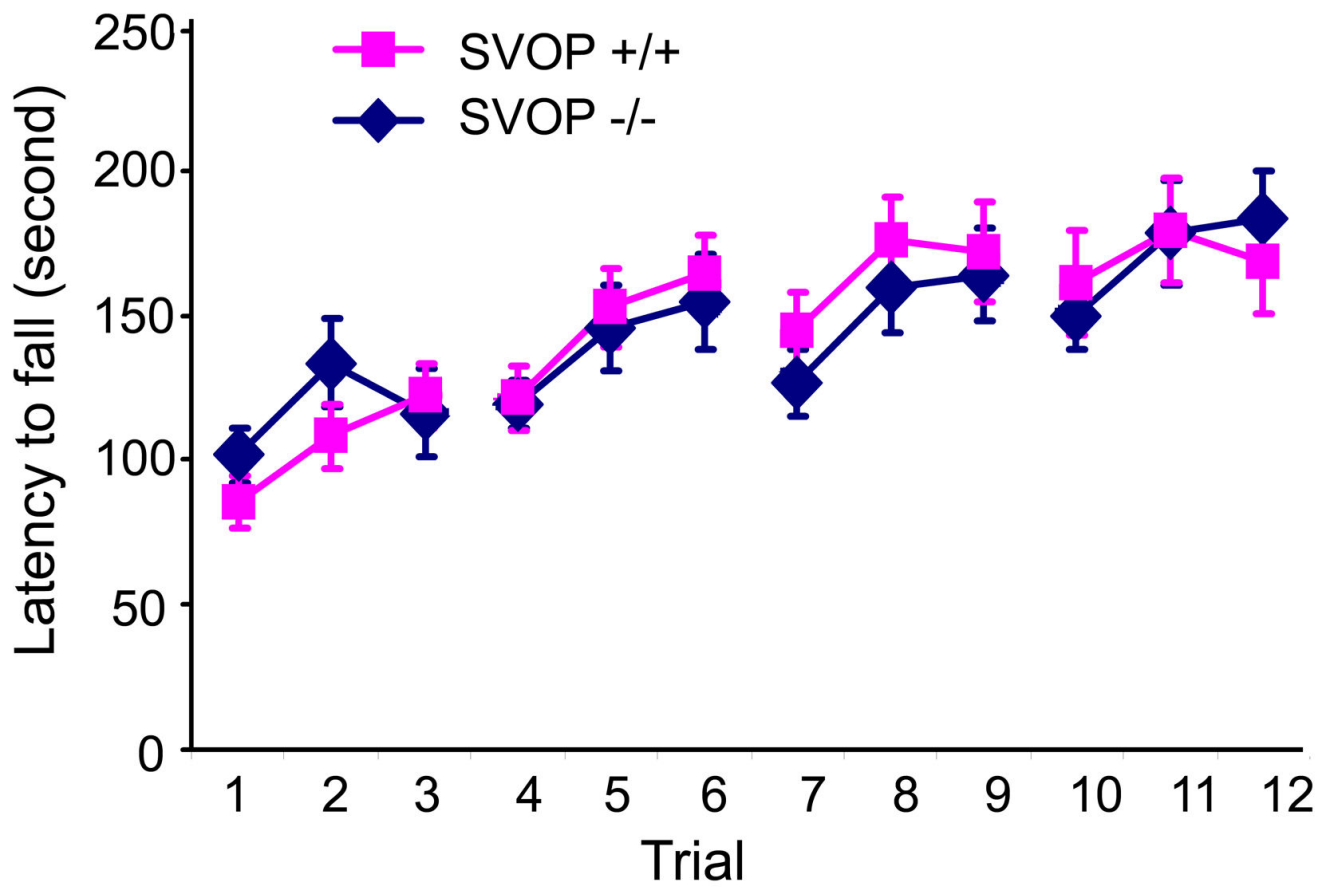
Loss of SVOP does not affect motor coordination. Mice were tested for motor coordination on the rotating rod. Twelve SVOP knockout (-/-) mice and 12 SVOP wild type (+/+) littermates were included in the test. Shown are average fall latencies across 12 trials (3 trials per day over 4 consecutive days). Each data point represents the group mean± SEM. Performance between groups did not differ (Students *t* test, *p* values ranged from 0.17 to 0.98 across trials, SAS software analysis (GLIMMIX) generalized linear mixed analysis of intra-individual correlation over time, *P*=0.90). Therefore, loss of SVOP did not affect motor performance.

The cerebellum plays an important role in motor control, and cerebellar deficits result in decreased motor coordination [27]. We assessed motor coordination in SVOP mutant mice by comparing their performance to wild-type mice on the rotating rod [27]. SVOP^-/-^ mice and age- and gender-matched wild type littermates, 8-10 weeks of age, were tested on an accelerating rotarod three times per day for four days. We observed no significant difference in baseline performance (first trial, latency to fall: SVOP ^+/+^ 85.5±8.6s, SVOP ^-/-^ 101.9±9.5s, *p*=0.22, two-tailed student *t* test) (Figure 6). Mice from both genotypes also demonstrated motor learning as indicated by increasing latency to fall across trials (GLIMMIX analysis of distributions, *p*=0.90). These results indicate that loss of SVOP does not impair cerebellar functioning or motor learning, consistent with SVOP being non-essential to neurotransmission in the cerebellum.

**Figure 7:**
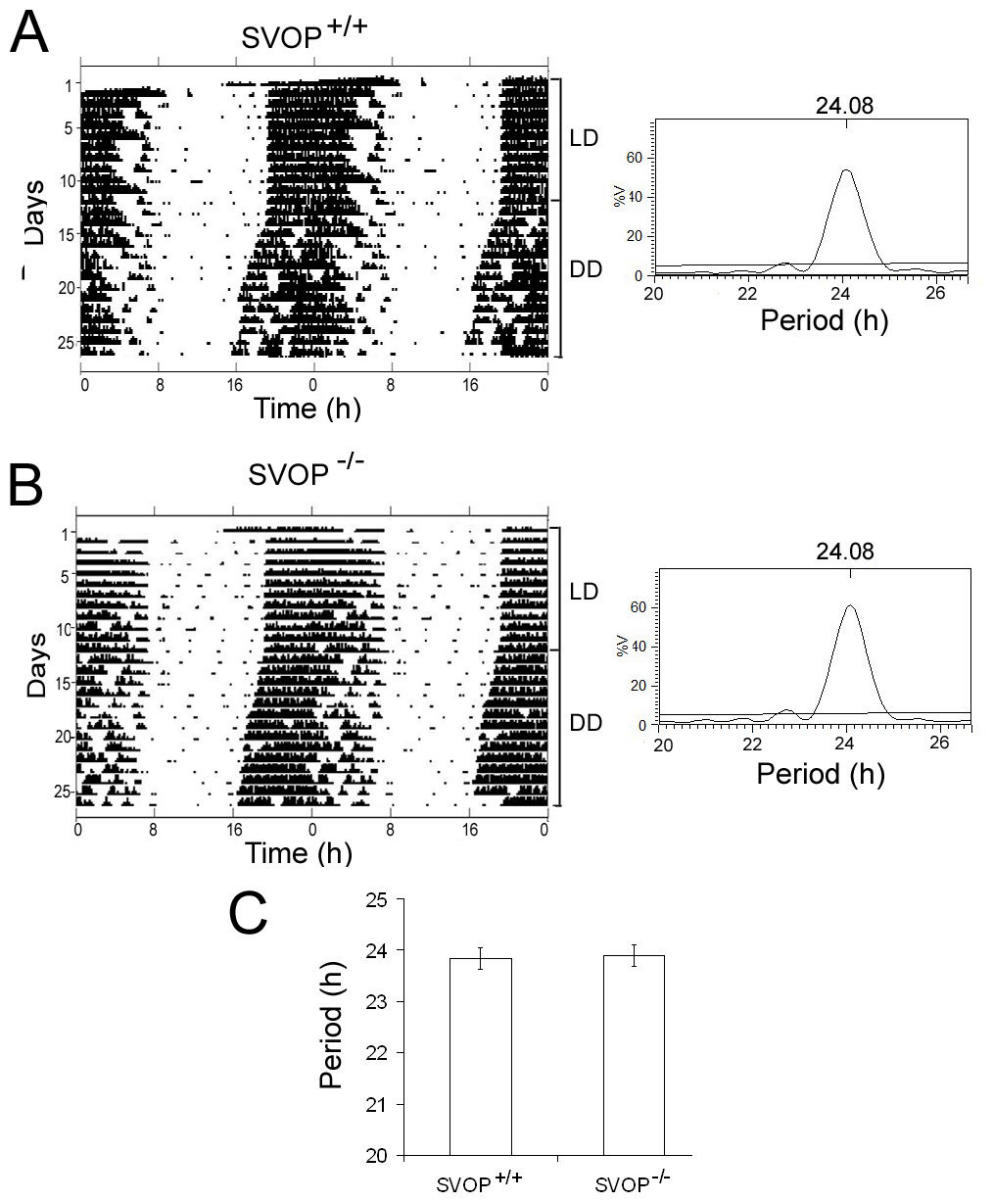
Circadian activity is normal in SVOP knockout mice. **A**, **B**) Shown are representative actograms and periodograms of wheel-running activity in wild-type (**A**) and SVOP knockout (**B**) mice under a 12 h : 12 h light/dark cycle and 12h: 12h dark/dark cycle. Left panels show representative actograms of two days’ activity plotted twice to demonstrate the circadian pattern. Right panels are periodograms that show the percent of behavioral variance (% V) accounted for by a circadian rhythm of *x* hours long. **C**) Calculated circadian period in constant darkness (DD) was similar for wild-type (+/+) and SVOP knockout (-/-) mice. The plot shows the mean ± SEM of the values calculated by periodogram analysis (two-tailed Student *t* test, *P* = 0.85, n = 6 for each genotype).

SVOP levels are also high in the pineal gland [6], which synthesizes and releases the hormone melatonin under the control of the suprachiasmatic nucleus (SCN) of the anterior hypothalamus, which houses the master circadian pacemaker of body. Several studies suggest that pineal melatonin also applies feedback regulation onto the SCN [28-30]. To look for effects of SVOP on circadian behavior, we compared activity in wild-type and SVOP mutant mice by monitoring running-wheel use throughout the day. The time dependent running of both wild-type and SVOP knockouts entrained to a 12: 12h LD cycle (Figure 7A, B). When transferred to total darkness (DD), wild-type mice displayed free-running circadian rhythms with a period of 23.85 ± 0.22 h. The free-running period of SVOP^-/-^mutant mice was indistinguishable from that of wild types (23.90 ± 0.20 h) (*p*=0.85, two-tailed student *t* test) (Figure 7B). Thus, loss of SVOP does not impair two key properties of the circadian system, its free-running period and its ability to entrain to LD cycles in mice (Figure 7C), suggesting that SVOP is not required for normal secretion of melatonin.

### SV2A/SV2B/SVOP triple knockout mice are phenotypically similar to SV2A/B knockouts

SVOP has been proposed to perform a function redundant to that of SV2. If true, loss of both gene families should result in a more severe phenotype than the loss of either one alone. To test this, we generated SV2A/SV2B/SVOP triple knockout mice. SV2B knockout and SVOP knockout mice are phenotypically normal. SV2B/SVOP double knockout mice also had no gross abnormalities. Crossing SV2A ^-/+^ SV2B^-/-^ SVOP^-/-^ mice produced pups of three SV2A genotypes at near Mendelian frequencies (61:118:60). The triple knockout mice demonstrated a phenotype no more severe than that of SV2A/B knockouts. Like SV2A/B knockout mice, they appeared normal at birth, but failed to grow, developed spontaneous seizures at approximately one week of age, and died within 3 weeks of birth. The lack of a more severe phenotype than observed with loss of SV2 alone suggests that SVOP does not perform a function redundant to that of SV2.

## Discussion

We report here that mice lacking SVOP are phenotypically normal. They demonstrate no differences in development, viability, fertility, seizure activity, major synaptic protein levels, synaptic transmission, motor coordination or circadian rhythms when compared to wild-type mice. Furthermore, mice lacking SVOP and the two major isoforms of SV2 (A and B) appear phenotypically identical to mice lacking SV2A and SV2B. Thus it appears that SVOP does not perform an essential function, or one that is redundant with SV2. Instead SVOP performs a function not apparent under the conditions assayed in our studies, suggesting that its role in nervous system functioning is subtle or specialized.

SVOP joins the ranks of apparently non-essential synaptic vesicle proteins like synaptophysin, another protein unique to synaptic vesicles [26]. We note that the abundance of SVOP protein in brain is low. By comparing standardized amounts of purified, epitope-tagged SV2 or SVOP to brain levels of these proteins using Western blot analysis, we estimate SVOP protein levels are less than 1% the level of SV2 (J. Yao, unpublished results). The fact that multiple proteomic studies of synaptic vesicle proteins failed to identify SVOP also speaks to the low abundance of SVOP [31-34]. The low level of SVOP is consistent with it performing a specialized, subtle function in the nervous system.

While our results suggest that SVOP does not perform a function redundant to that of SV2, it remains possible that loss of SVOP function is compensated by another protein. The most likely candidate is SVOP-like protein (SVOPL) [6]. SVOPL shares 39% amino acid identity to SVOP and is also expressed in brain [6].

In studies of rodent and human mRNA expression during aging, SVOP mRNA decreased significantly with aging [18,19]. To determine if this was true of SVOP protein expression, we compared brain SVOP protein levels in mice at 10 months (n=6), 20 months (n=2), and 28 months of age (n=4). Our preliminary results showed a significant decline, suggesting that decreased SVOP protein expression accompanies brain aging. We found, however, no evidence of an early aging phenotype in the SVOP knockouts. For example, both fertility and rotarod performance decrease with aging [35-38], yet we did not see an early deficit in either in SVOP knockout mice. Therefore our findings also suggest that loss of SVOP expression does not, by itself, produce brain aging, though we note that the behavioral studies reported here were done with mice ≤ 4 months old. It thus remains possible that a phenotype would emerge later. Another possibility is that the age-related decline in SVOP expression provides protection against the effects of aging, which would not be apparent in younger mice.

In conclusion, our findings support the interpretation that SVOP performs a function that can be compensated for under normal conditions. Future studies of synaptic vesicle protein networks will likely elucidate how each protein contributes to synaptic functioning.
